# How do Employees with Chronic Musculoskeletal Disorders Experience the Management of Their Condition in the Workplace? A Metasynthesis

**DOI:** 10.1007/s10926-023-10099-2

**Published:** 2023-02-27

**Authors:** Glykeria Skamagki, Christine Carpenter, Andrew King, Charlotte Wåhlin

**Affiliations:** 1 Department of Physiotherapy, School of Sport, Exercise and Rehabilitation, University of Birmingham, Birmingham, UK; 2 Department of Physiotherapy, University of British Columbia, Vancouver, Canada; 3 Department of Physiotherapy, Coventry University, Coventry, UK; 4 Department of Health, Medicine and Caring Sciences, Occupational and Environmental Medicine Center, Division of Prevention, Rehabilitation and Community Medicine, Unit of Intervention and Implementation Research, Institute for Environmental Medicine, Linköping University, Karolinska Institutet, Stockholm, Sweden

**Keywords:** Chronic musculoskeletal diseases, Workplace, Qualitative research, Management

## Abstract

This metasynthesis contributes to an understanding of the experiences, perceptions, and attitudes of employees on managing chronic musculoskeletal disorders (CMSDs) at work. Many studies in this field are concerned with prevention or return-to-work (RTW) programmes. However, the purpose of this review was to synthesise evidence that only focuses on the employees’ management of their CMSDs at work. The SPIDER framework was used to structure the question “How do employees with CMSDs experience the management of their condition in the workplace”? The literature search focused on articles published between 2011 and 2021, and the search was conducted using the following databases: MEDLINE, SCOPUS, CINAHL, AMED, PsycINFO. The review identified nine articles that explored employees’ experiences of managing CMSDs at work. Thematic synthesis was used to create analytic themes which provided a more in-depth discussion of these experiences. The identified themes were: ‘employees actively seek ways to manage their conditions’, ‘influence of work environment on employees with CMSDs’ and ‘optimising the relationship between employees and managers. This metasynthesis suggests that the ability to negotiate workplace support and manage CMSDs at work is influenced by the cultural and social environment of the organisation. Effective communication, care and trust between the employee is needed. The review also illustrated the need for healthcare professionals to provide support to employees at work.

**Supplementary Information:**

The online version contains supplementary material available at 10.1007/s10926-023-10099-2.

## Introduction

Chronic musculoskeletal disorders (CMSDs) are a major cause of work disability with severe consequences on the working ability and prolonged employability of older employees [1–3]. They present a challenge due to the associations between sick leave rates, ‘presenteeism’ and reduced productivity levels [4, 5]. CMSDs are characterised by a generally slow progression that requires continuous and long-term management [6]. People with CMSDs may develop more than one chronic condition in their lifetime such as cardiovascular diseases, diabetes or depression [7, 8]. Public Health England [9] indicated that 13% of those aged 18 reported at least two chronic conditions, of which one is a CMSD. Multimorbidity is a factor that illustrates the complex needs of the workforce. Working may bring financial, psychological and physical benefits, however, employees with CMSDs may struggle with the demands of their work, the ageing process and the management of multimorbidities [10–13].

Recent reviews [14–18] investigated the effectiveness of work interventions to manage these conditions. The studies concluded that physical activity programs and/or integrated healthcare delivered in the workplace are effective in supporting the management of musculoskeletal disorders (including CMSDs). However, these studies evaluated programmes which were not based on established workplace interventions, were heterogenous and were conducted in different countries. As employees do not have access to standardised systems of support at work it is important to explore the experiences of those managing CMSDs at work.

The initial scoping search identified primary studies, meta-synthesises and meta-ethnographies exploring the management of chronic musculoskeletal pain. However, some of these studies synthesised primary research that had been conducted in the decade before 2012 [19–21] that did not address the question developed for this review, aimed to develop a conceptual understanding of living with chronic non-malignant pain [22] or chronic pain in general [23] or focused on experiences after experimental trials, return-to-work programs or evaluated self-management interventions [24, 25].

This metasynthesis aims to contribute to a better understanding of the experiences, perceptions, and attitudes of employees on managing chronic musculoskeletal disorders CMSDs at work. As studies that focus on the management of musculoskeletal health in the workplace remain relatively overshadowed by prevention or return-to-work strategies it was important to synthesise evidence with a focus only on management. The SPIDER framework (Table 1) was used to structure the question “How do employees with CMSDs experience the management of their condition in the workplace”? The SPIDER tool facilitates rigour in research by defining key elements of qualitative research questions [26].

**Table 1:** Use of the SPIDER Framework to create a qualitative review question

**Setting/Sample**	Any workplace environment/employees with CMSDs
**Phenomenon of Interest**	Experience of any work strategy offered and/or used to manage CMSDs
**Design**	Interviews, focus groups
**Evaluation**	Experiences, attitudes, perspectives
**Research type**	Qualitative studies, mixed methods research studies

## Methods

A metasynthesis (systematic review and integration of findings from qualitative studies) involves a comprehensive critical interpretation of the literature that may identify gaps or inconsistencies and provide a better understanding of the topic of interest [26]. Ethical approval for this review was granted by Coventry University Ethics committee.

### Search Strategy

A systematic search was conducted to identify all the qualitative studies that focused on the experiences, perceptions, and attitudes of employees with CMSDs towards the management plans offered at work. The criteria used to include and exclude studies are reported in Table 2.

**Table 2:** Inclusion and Exclusion criteria

Inclusion criteria	Exclusion criteria
Qualitative research and qualitative components of mixed methods research studies published in peer-reviewed journals that explored adult employees’ experiences of managing chronic non-malignant musculoskeletal pain at work.	Studies that investigated workplace interventions that exclusively focused on injury prevention or return-to-work. Studies that explored acute MSDs, neurological pain [e.g. stroke, multiple sclerosis], dental, menstrual pain, or other serious pathologies.

The literature search focused on articles published between 2011 and 2021, and the search was conducted using the following databases: OVID MEDLINE (1946–2021), ELSEVIER SCOPUS (2004–2022), EBSCO CINAHL (1937–2022), EBSCO AMED (1985–2022), OVID PsycINFO (1806–2021) (example provided). A combination of free-text terms and thesaurus terms or subject headings were generated to access relevant qualitative studies. Search strategies used Boolean operators (AND/OR/NOT), subject headings, different spellings, acronyms, and wild cards. The reference lists of the included articles were also checked to potentially identify qualitative studies that may have been missed from the original search. The literature search was conducted between February 2021 and September 2021 as articles before 2012 did not match the developed research question. The search was conducted by lead researcher and reviewed by the second author. The papers included were published in English in peer-reviewed journals. Lastly, SCOPUS was used to identify articles that have been cited more recently but were missed from the database search.

### Screening

**Fig. 1:**
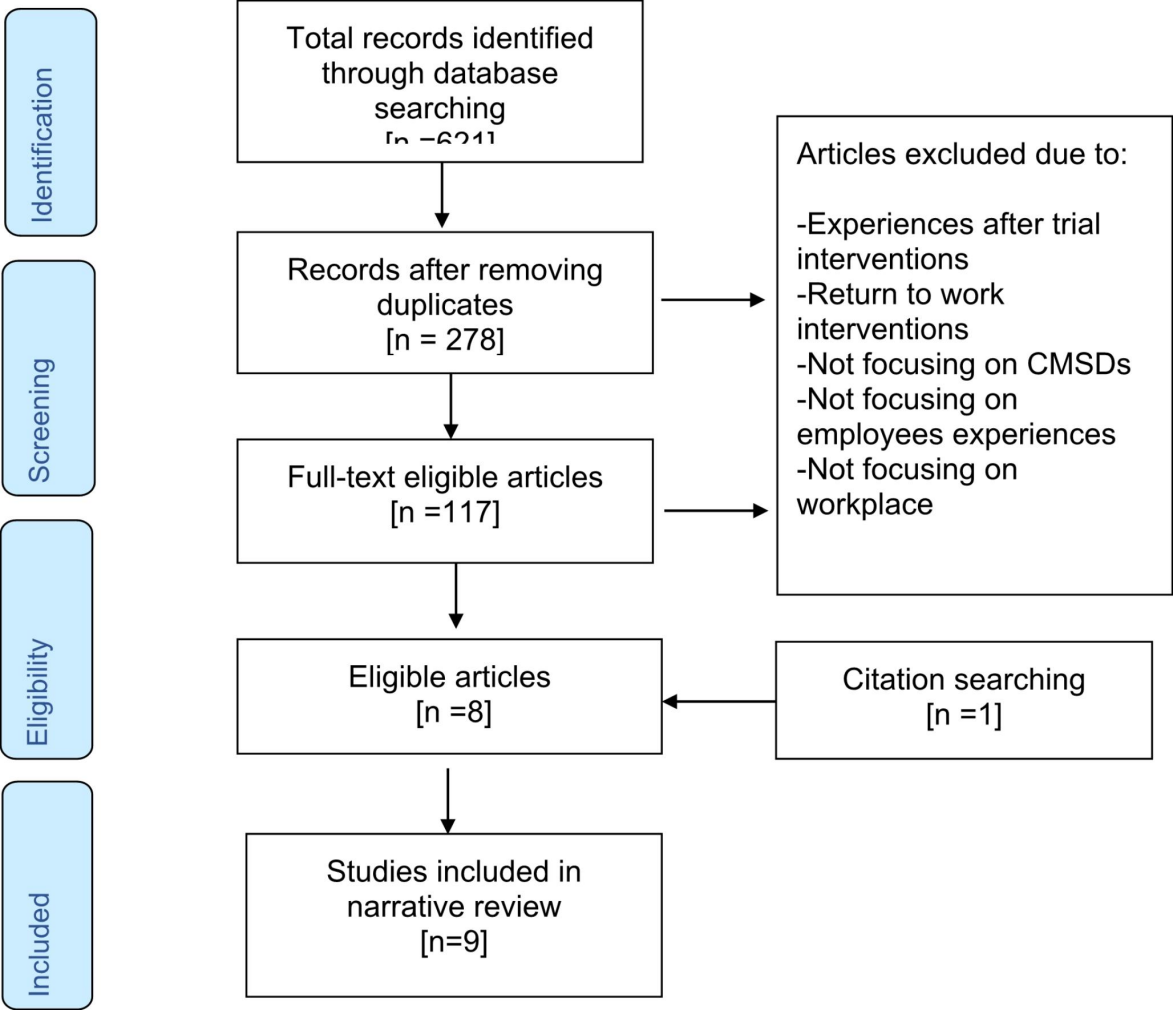
PRISMA Flow chart of study identification, selection and synthesis

The search identified a total of 621 references: 12 references in AMED, 165 in Academic Search Complete, 231 in MEDLINE, 144 in CINAHL and 81 in PsycINFO. The articles were screened by the first and second author based on their title, abstract, study design and relevance. After removing duplicates and refining the search results, 9 articles remained (Fig. 1). The key characteristics of the included papers are outlined in Table 3.

**Table 3:** Summary Table of Reviewed Literature and their characteristics

Authors	Country	Methodology and Methods	Participants	Data analysis	Themes
Oakman et al. [31]	Australia	Phenomenology Semi-structured interviews	N = 40, Age range: 18+ Manual and Sedentary occupation	Thematic analysis	Meaning of work, disclose or not, information seeking, gaps in resources, trusted sources
Agaliotis et al. [37]	Australia	Not specified Focus groups	N = 17 Age range: 51–77 Employees with chronic knee pain Private and public sector Professional and manual occupation	Systematic analysis used in grounded theory approach	The effect of knee pain on work productivity, strategies to improve work productivity, future suggestions about sustainable work
Oakman, Kinsman, and Briggs [30]	Australia	Mixed Methods Research Questionnaire followed by semi-structured interviews	N = 35 Age range: 25+ Adult employees with chronic musculoskeletal pain Private and public sector	A thematic approach using grounded theory principles.	Barriers to working productively, enablers to working productively, disclosing my condition at work
Holland and Collins [36]	UK	Not specified Semi-structured interviews	N = 11 Age range:32–58 Adult employees with rheumatoid arthritis Self-employed, private and public sector. Professional and semi-skilled occupations	Thematic analysis	The perceived importance of work, seeking normality after first onset, keeping productive, and employed through workplace adjustments, sickness absence policies causing pressure to work
Kalsi et al. [32]	UK	Not specified Focus groups	N = 17 Age range: 18–34 Adult employees with chronic pain	Thematic analysis	Living with chronic pain. the fine balance between chronic pain and return to work, work is a beautiful thing when you have it, the luck of the draw
Hutting et al. [33]	Netherlands	Not specified Focus groups [and three individual interviews due to participants’ attendance]	N = 15 Age range:25–56 Adult employees with complaints of the arm, neck, or shoulder. Professional and semi-skilled occupations Private and public sector	Convention-al content analysis	Ideas about the causes of complaints, dealing with non-visible complaints, experiences with different forms of treatment, workplace adjustments
De Vries et al. [35]	Netherlands	Not specified Semi-structured interviews	N = 21 Age range:30–60 Adult employees with chronic musculoskeletal pain Professional, unskilled and manual occupation Self-employed, private and public sector	Thematic analysis	Motivators to stay at work, Success factors for staying at work
Coole, Watson, and Drummond [34]	UK	Not specified Semi-structured interviews	N = 25 Age range: 22–58 Adult employees with low back pain. Self-employed, private and public sector from large and medium-sized companies. Professional, unskilled and manual occupation	Thematic analysis	Occupational Health assistance, assistance from employers/managers, work modifications and patient control
Wynne-Jones et al. [29]	UK	Mixed Methods Research Questionnaire and the semi-structured interviews	N = 18 employees Mean: 49,7 Adult employees and managers with chronic musculoskeletal pain Public sector Wide range of occupations	Thematic analysis	Impact of health, moral aspects of absence and attendance, absence management policies and return to work

### Quality Appraisal

It is essential to assess the quality of any published research before trusting its findings. For the purpose of this review, the Joanna Briggs Institute Critical Appraisal tool (JBI-QARI) was selected to guide appraisal of the included studies [27, 28] as it provides congruency and it is coherent and clear in relation to questions about the philosophical perspective, methodology, and study design. A summary of the critical appraisal of the reviewed articles using the JBI-QARI is provided in Table 4 and the framework can be found as a supplementary document at the end of the article. The studies were reviewed by the lead author and double reviewed by the second and third author. The qualitative components of the two mixed methods research studies included in the review [29, 30] were of moderate quality, whereas the quality of six qualitative research articles was assessed as poor to moderate. Only one qualitative study was assessed as high quality [31].

**Table 4:** Critical appraisal of included studies using the Joanna Briggs Institute Critical Appraisal tool

Authors	Q1	Q2	Q3	Q4	Q5	Q6	Q7	Q8	Q9	Q10	Overall appraisal
Oakman et al.[31]	Y	Y	Y	Y	Y	N	N	Y	Y	Y	No concerns about the design. Clear description throughout.
Agaliotis et al. [37]	N	Y	Y	U	U	N	N	N	Y	U	Limited information on methodology and theoretical perspectives. Participants’ voice and researchers’ position is not adequately represented. Limited illustrative quotes.
Oakman et al. [30]	Y	Y	Y	Y	U	N	U	U	Y	Y	Moderate description of methods and analysis of the results. Information given but not in great detail. Unclear about pilot testing.
Holland and Collins [36]	N	U	U	U	U	N	N	Y	Y	Y	No information on methodology and theoretical perspectives. Moderate description of methods and analysis of the results.
Kalsi et al. [32]	N	U	U	U	U	N	N	Y	Y	U	No information on methodology and theoretical perspectives. Moderate description of methods and analysis of the results.
Hutting et al. [33]	N	N	U	U	U	N	N	U	Y	U	No information on methodology and theoretical perspectives. Limited description of methods and analysis of the results. Questionable member checking [a year after the focus group]. Concerns about the design of the study.
De Vries et al. [35]	N	U	U	U	U	N	N	Y	Y	Y	No information on methodology and theoretical perspectives. Moderate description of methods but limited understanding of qualitative research.
Coole, et al. [34]	N	U	U	U	U	N	N	Y	Y	Y	No information on methodology, theoretical perspectives and researchers’ position and influences. Moderate description of participants’ recruitment.
Wynne-Jones et al. [29]	Y	Y	Y	Y	Y	N	N	Y	Y	Y	Moderate description of MMR methods.

Note: Q=Question, Yes=Y, N=No and U= Unclear

While no studies were excluded on the grounds of quality due to the potential risk of losing valuable insights, the reader should be aware that not all studies included were of the same methodological standard. None of the authors, with the exception of two [29, 30], discussed the methodology on which their studies were based. All the qualitative studies but one [31] failed to show congruity between the stated methodology and the study aim and objectives. On the other hand, the MMR studies gave sufficient detail about MMR as a methodology and the design elements.

Two articles [32, 33] did not include a discussion of the role of the researcher in designing and implementing the study or of the study limitations. As a result, it was not possible to judge the degree to which the researcher or other factors influenced the study design and analysis process. For example, in one study [33], participants who could not attend the focus groups were offered the option of an individual interview. However, the researchers did not explain this decision, for example, how it was implemented, or the challenges faced. In addition, the authors who did discuss the study limitations did not demonstrate a thorough understanding of qualitative research. For example, Coole et al. [34] and De Vries at al. [35] discussed their study findings in terms of ‘generalisation’, which is a quantitative concept that is generally considered not appropriate or achievable in qualitative research .

As the qualitative studies did not sufficiently justify the choice of data collection methods, it was difficult to assess whether the methods chosen provided the best fit. Similarly, none of the authors discussed the researchers’ roles and responsibilities in the research or how their interests in the research topic and participants may have influenced the decisions taken in the study design and the interpretation of the findings. In addition, none of the study reports provided details about the participant recruitment process and the development of the interview guide.

For example, Oakman, Kinsman and Briggs [30] explained that they changed the interview questions. It was unclear why the authors included this information or why the questions needed to be changed after two participants were interviewed. It would have been useful to know whether these questions constituted a preliminary pilot test of the proposed questions or an ongoing development of an interview guide, as the latter strategies can be useful for less experienced researchers. Details about the participants’ characteristics were also missing. For example, sample size decisions were not justified by the authors except in two studies [31, 34] and the participant age range varied between over 18 [31], over 20 [30, 32–34] over 30 [35, 36] and over 50 [37]. Only two studies [31, 35] provided detailed information about the participant recruitment process and interview preparation.

Participants’ quotes were limited or missing in three studies [30, 33, 37] which undermined the ‘credibility’ of the interpretation and representation of the findings. As a result, it was difficult to determine if the participants’ voices were adequately represented in these studies. In addition, these authors did not provide a transparent description of the steps taken in analysing and interpreting their respective data. All the studies reviewed provided an outline of the study strengths and limitations. The qualitative components of the two mixed methods research studies included in the review [29, 30] were of moderate quality, whereas the quality of the six qualitative research articles was assessed as poor to moderate.

### Synthesising the Findings

Thomas and Harden’s [38] approach to the synthesis of qualitative research findings [thematic synthesis] was used to guide the analysis of this review. Each paper was carefully reviewed, and relevant information was extracted. The review was led by first author and double reviewed by the third and the fourth author. The selected findings were uploaded onto NVivo 13 allowing the creation of memos, codes and subthemes. Several conceptual maps and diagrams were created to elevate the descriptive themes and ‘go beyond’ the findings of the primary studies and generate the final ‘analytic themes’. The list of final themes and subthemes are presented in Table 5.

**Table 5:** List of Analytical themes and subthemes

**Employees actively seek ways to manage their conditions**
Occupational health
Personal strategies
Changes in the job status
**Influence of work environment on employees with CMSDs**
Colleagues
Manager
Employer
**Optimising the relationship between employees and managers**
Care
Trust
Communication

## Results

Nine articles that explored employees’ experiences of managing CMSDs at work were reviewed. Participants were both males and females [more females than male] recruited from both professional and semi-skilled occupations in either private or public sector organisations. Age varied between 18 and 77 years old. It is important to mention that the verbatim findings of the studies used the terms ‘employer’, ‘manager’, and ‘organisation’ mainly interchangeably.

### Influence of Work Environment on Employees with CMSDs

Participants in the included studies had experienced both supportive and unsupportive behaviours from employers, and managers, but a number of more negative experiences were consistently discussed. Some managers were perceived as being unhelpful and showing a limited understanding of the employees’ needs. In contrast, managers who had themselves experienced a musculoskeletal problem were more helpful [34, 36]. It was clearly important to employees that managers could recognise the impact of a CMSD and approve adjustments or facilitate some flexibility at work [34, 36]. For example, one participant in Holland and Collins’ [36] study explained that the manager allowed her to change her working hours when she was not feeling well and, in that way, she was able to maintain her productivity levels and successfully manage her flare-ups.

Some studies reported that employees found managers generally unsupportive and difficult to work with [29–31, 34, 37]. In one study, participants shared examples of managers micromanaging their breaks and their time away from their desks [34] and in another study unsupportive environments were perceived as a barrier for some participants to disclose their condition [31]. Finally, some studies reported that managers had refused to provide workplace adjustments or had decided that employees did not need them without further discussion [29, 30, 34, 37]. For example, in one study [37], a participant reported that the manager refused to provide the suggested work adjustments due to the ‘invisible’ nature of employee’s symptoms.

On the whole, employees in the selected studies were working in an organisation that offered access to health services, e.g. occupational health assessments or physiotherapy, but this access was intended only to support a return-to-work process and not the long-term management of the condition [29, 34]. However due to the heterogeneity of the included studies the size of the organisation and the nature of the job cannot be discussed. Employees expressed, that in their experience, employers lacked knowledge and information or resources to support the development of improved working conditions [31]. Some authors reported resistance from employers when employees requested flexible work hours and workplace modifications [32, 34]. For example, a participant in Coole et al. ‘s [34] study explained that the employers in the UK were not prepared to fund the recommended ergonomic equipment.

A few studies reported positive experiences from co-workers who either helped employees with their tasks [34] or showed understanding when employees were unwell and could not perform physical job tasks adequately [37]. Some employees described instances where they were not believed by their work colleagues [33] or they identified obstacles due to the work culture or the excessive workload [29].

### Employees Actively Seek Ways to Manage Their Conditions: Personal Strategies

This theme highlighted those employees who were keen to take responsibility for managing their CMSDs at work and identified some of the strategies they used. Employees described personal strategies used for example online resources or visiting allied health professionals [30, 31, 33–37]. In general, employees found the professional advice they received to be effective in assisting them to make adjustments at work but the adjustments described varied considerably across the studies reviewed. In addition, employees explained that the culture of the organisation and the nature of a job could also affect the management of CMSDs [30, 32–37].

The majority of the reviewed studies illustrated that pain relief medication allowed employees to work better and reduced the need to take frequent or long-term sick leave [32–35]. It is however unclear whether positive outcomes discussed were as a direct result of the medication or if employees had combined them with other interventions. Some of the reviewed studies illustrated that employees were reluctant to take medications due to their side effects [30, 39]. If there are no other interventions to support employees in managing their condition, then the use of medications may have adverse effects on their work abilities and further exploration of this issue is needed.

Findings highlighted a positive input from the healthcare professionals [HCPs], i.e. physiotherapist, occupational therapist [30, 31, 33–35]. It is important to note that some study participants were employees participating in RTW programs and had access to an on-site occupational health service [OHS] or worked in organisations that offered private healthcare services. Some studies [30–33] highlighted that HCPs were able to effectively explain the nature of CMSD and the impact at work or provide them with educational resources. However, in most of the studies, no explanation of how employees were referred or obtained an appointment for these services was provided [30, 35, 36, 40].

Lastly, findings illustrated that some participants felt the need to change jobs in order to manage their CMSD more effectively [30, 31, 33, 34, 37, 39]. Some findings suggested that the negative impact the CMSDs had on employees’ mental and physical health was reduced when they changed jobs [33, 35]. Other authors reported that employees who experienced reduced work ability preferred part-time work [30, 33] or had chosen jobs which minimised high physical demands and enabled flexibility [31]. In addition, becoming self-employed was a preferred choice in studies where employees felt unsupported in their workplace [34, 35].

### Optimising the Relationship Between Employees and Managers: Communication

Studies included in this review explained how ‘effective’ communication mainly related to the managers’ willingness [or not] to discuss employees’ circumstances [29–31, 37, 40]. Five of the included studies discussed managers’ perceived duty of care to employees with CMSDs [29, 30, 32, 36, 41]. The impression in some studies was that managers who lacked interest, experience or understanding of CMSDs were perceived to have a negative impact on how employees managed the condition, e.g. by ignoring useful advice provided by OHS or not knowing where to find appropriate resources [29, 31, 32, 36, 41].

Finally, some studies illustrated the importance of creating an environment of trust between employees with CMSDs and managers [29, 34, 36]. These studies highlighted that not all managers had the employees’ best interests in mind when offering support strategies. Study authors identified several reasons why the important relationship between the manager and employee could deteriorate: for example, strict sick leave policies that result in employees’ feeling that their job stability was threatened if they failed to RTW in the timeframe outlined.

## Discussion

This qualitative review indicates that the workplace environment may influence the support offered to employees with CMSDs. It highlights that the work environment is affected by the behaviours and beliefs of the employer, manager and co-worker. The findings reinforce that access to healthcare services may enhance effective communication and trust between the employee and manager influencing the support provided to employees.

Managers were perceived as supportive only when they understood the condition, provided ergonomic adjustments and offered flexibility. The review highlighted that the nature of the support differed from one manager to another; thus educational opportunities and training about the nature of CMSDs and the impact they have at work would be useful to consider. Current research also suggests that managers, who are experienced by employees as inflexible and unsympathetic, are an obstacle to the RTW process [42] or the management of CMSDs [43]. Toye et al. [21] suggested that a supportive work environment may enable employees with CMSDs to better manage the impact of their condition at work. However, as there is no standard approach to supporting employees with CMSDs in the workplace, organisations need to be encouraged to take a more dynamic role and develop sustainable management plans for those employees.

The included studies illustrated that workplace with access to an OHS or private healthcare appeared to support employees better after they had been on long-term sick leave. However, it was unclear in this review how accessible these services were as only some workplaces offered access to an OHS or to private HCPs who could support these employees. For example, healthcare professionals can create plans tailored to an employee’s needs that include supportive and meaningful strategies and assist them to stay at work longer [44–46]. Professional advice would appear to be an essential element in designing an appropriate management programme for employees with CMSDs [18, 47–49]. The main focus of occupational health and safety (OSH) services is on protecting and supporting the health and well-being of the workforce and further research that explored the experiences of those employees who do not have access to OHS at work would be useful.

Findings in this review suggested that professional recommendations were not always taken into consideration by organisations who delivered occupational health services. Therefore, employees felt that adjustments to duties, working hours, or ergonomic equipment had to be constantly negotiated and, on occasion, were refused. It is important to understand and mitigate the safety implications of work arrangements and developed proactive systems that can protect workers, prevent injury and manage illness, and promote well-being. However, the barriers and facilitators associated with the implementation of OHS recommendations in the workplace to support the management of a CMSD have not been comprehensively studied. For example, RTW and management initiatives could develop a dynamic and individualised process that included employees’ experiences prior to and following work resumption [50]; however, this discussion has, to date, mainly occurred at a theoretical level [51].

Research that focused on the prevention of musculoskeletal disorders suggests that financial constraints, reduced resources and a general lack of organisational awareness about MSDs act as barriers to the implementation of ergonomic advice [52–54]. Our review supported these findings [52, 54–56] and suggested that it would be useful if relevant resources could be consistently made available to facilitate employees’ access to professional healthcare services. No other strategies or specific interventions, with the aim of assisting employees to effectively manage their conditions, were identified. As the nature of the workplace and workforce changes work-based interventions provided by an OHS, wellbeing initiatives or online services need to be rigorously explored. Services that support the provision of healthcare services at work could potentially make a positive contribution to sustaining employees’ health.

This review suggested that older employees with CMSDs were thinking of changing their job role and status due to the impact their condition had on their work. A review [57] of the economic and productivity factors related to the management of MSDs by the workforce in Europe reported that, because work ability varied amongst employees with a CMSD, many of them did not perform to their full capacity. Chronic musculoskeletal disorders can impact employees’ working lives differently. For example, older employees in the same review, who were thinking of early retirement, preferred to become unemployed without sufficient financial support [57] due to the progression of their condition. Ongoing changes in the retirement policies and challenges in the primary healthcare have made exploration of how the ageing workforce manages CMSDs at work more imperative.

Employees in this review were keen to take responsibility for the management of their condition. However, the personal strategies identified were limited in number, scope and were not always congruent with the dimensions that characterise self-management interventions for chronic conditions. Self-management interventions may enable employees to participate actively and take control of their chronic condition [58]. A greater understanding of how different interventions can assist employees in addressing the personal, psychosocial, and biomechanical components of managing a CMSD and how they could be effectively promoted in the workplace would be useful [59–61].

Finally, this review has reinforced that the relationship between employees and their managers/employers directly impacts employees’ abilities to manage their condition. Values such as trust, care and communication are important to employees. Smith and Brunner [62] explored how people decide to disclose their condition at work. Their findings revealed that the organisational culture [including managers’ attitudes] shaped the environment for or against disclosure. For example, the authors found that building trust and educating others about the conditions would positively influence disclosure. Therefore, studies that seek to explore the values that underpin the employee-manager relationship and its impact on health and work would contribute to our understanding of the issues involved from the manager and employee’s perspectives.

### Implications for Rehabilitation and Research

Workplace programmes and current research mainly focuses on the RTW process and largely neglects the sustainable management of CMSDs after employees return on full duties.

The roles of healthcare professionals, managers and co-workers in supporting employees with CMSDs at work are key but further research is needed to contribute to the development and promotion of supportive work cultures.

Overall, despite the changes in demographics and retirement policies in many countries, a research gap has been identified in relation to the experiences of older employees and their abilities to manage CMSDs at work.

### Limitations of the Review

his review included nine articles reporting qualitative research studies conducted in Australia, The Netherlands and the United Kingdom that provided a number of insights about the experiences of employees with CMSDs. There are considerable differences in these countries’ national health and social services and these may limit potential transferability of some of the findings. The majority of the participants in the included studies were females in various age groups which did not provide a good age coverage and a decrease the richness of the data obtained for each age range. Lastly, minimum information on employment practices and work settings further limits the transferability of the findings.

The studies included in this review, based on the information provided by the authors, were generally not of a high methodological standard. The authors did not provide adequate information about the chosen methodology, participants’ recruitment process, nor were the data collection methods and analysis explained in any detail. There was no indication of any reflective practice or discussion of the potential influence the researcher may have on the research process. Lastly, the included studies rarely provided sufficient illustrative participant quotes to support the authors’ findings.

## Conclusion

Rigorous qualitative research and mixed methods methodologies have much to contribute to our understanding of the complex and multifactorial issues that impact the experience of employees who manage a CMSD. This review illustrated the importance of the work environment, the social components of work, and the employee-manager relationship in supporting employees with CMSDs. The role of occupational health services and the interventions used may provide support to employees with CMSDs.

### Electronic Supplementary Material

Below is the link to the electronic supplementary material.

Supplementary Material 1

Supplementary Material 2
