# Supplementary material for: How do Employees with Chronic Musculoskeletal Disorders Experience the Management of Their Condition in the Workplace? A Metasynthesis
Source: J Occup Rehabil. 2023 Feb 27;33(4):702–12. doi: 10.1007/s10926-023-10099-2 (PMC10684637; doi:10.1007/s10926-023-10099-2)
Supplement: Supplementary file 1 — Supplementary Material 1 [file 10926_2023_10099_MOESM1_ESM.docx]

Supplementary document showing a search strategy example in CINAHL Database (10/05/2021). Combinations that produced zero results are not depicted in the table.

| **Search ID#** | **Search Terms** | **Results** |
| --- | --- | --- |
| S1 AND S2 AND S4 AND S5 AND S6 AND S7 |  | 183 |
| S1 AND S3 AND S4 AND S5 |  | 1.527 |
| S1 AND S2 AND S4 AND S5 AND S6 AND S7 AND S9 |  | 1 |
| S1 AND S2 AND S4 AND S5 AND S6 AND S7 AND S10 |  | 48 |
| S1 AND S2 AND S3 AND S4 AND S5 AND S6 AND S7 |  | 181 |
| S10 | "qualitative research" OR "qualitative study" OR "qualitative method*" OR interview* OR "focus group*" | 376.935 |
| S9 | "mixed methods research" OR "mixed methods research | 10803 |

|  | design" OR mixed methods" OR "mixed approach*" OR "mixed methodology*" |  |
| --- | --- | --- |
| S7 | condition* OR disease* OR disorder* OR "ill health" OR pathosis OR illness* OR complaint* OR pain | 2.220.144 |
| S6 | "chronic" or "long-term" | 511.368 |
| S5 | manage* OR cope* OR decrease* OR improve* OR control* OR handle* OR avoid* OR reduce* OR stop* OR "deal with" | 2.576.261 |
| S4 | employee* OR worker* OR workforce OR "work force" OR staff OR personnel OR "labor force" OR laborforce OR "labour force" OR "labour force" OR work OR workplace OR job OR occupation | 794.703 |
| S3 | (MM "Musculoskeletal Diseases+") | 222.900 |
| S2 | "chronic musculoskeletal disorder*" OR "musculoskeletal condition*" OR MSD* OR MSDs OR  "musculoskeletal pain" OR inflammatory conditions OR “inflammatory disorder*” OR "chronic musculoskeletal condition*" "musculoskeletal disorder*" | 20.130 |
| S1 | belief* OR opinion* OR attitude* OR viewpoint* OR perspective* OR thought* OR  expectation* OR experience* | 1.109.482 |

|  | OR view* OR behavio?r OR idea* OR feeling* |  |
| --- | --- | --- |
