# Supplementary material for: How do Employees with Chronic Musculoskeletal Disorders Experience the Management of Their Condition in the Workplace? A Metasynthesis
Source: J Occup Rehabil. 2023 Feb 27;33(4):702–12. doi: 10.1007/s10926-023-10099-2 (PMC10684637; doi:10.1007/s10926-023-10099-2)
Supplement: Supplementary file 2 — Supplementary Material 2 [file 10926_2023_10099_MOESM2_ESM.pdf]

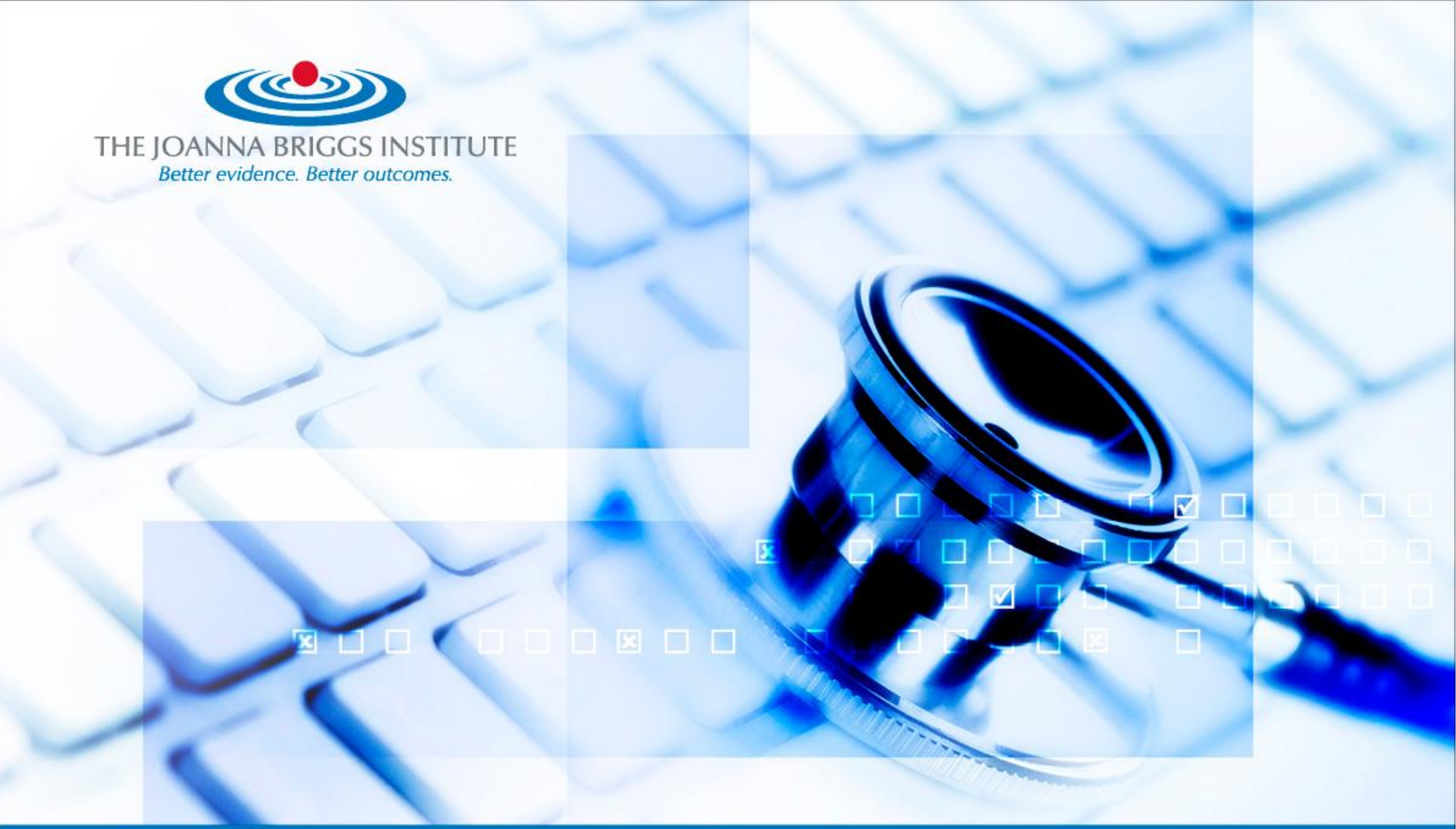

THE JOANNA BRIGGS INSTITUTE
Better evidence. Better outcomes.

The Joanna Briggs Institute Critical Appraisal tools
for use in JBI Systematic Reviews

Checklist for Qualitative Research

http://joannabriggs.org/research/critical-appraisal-tools.html

THE JOANNA BRIGGS INSTITUTE

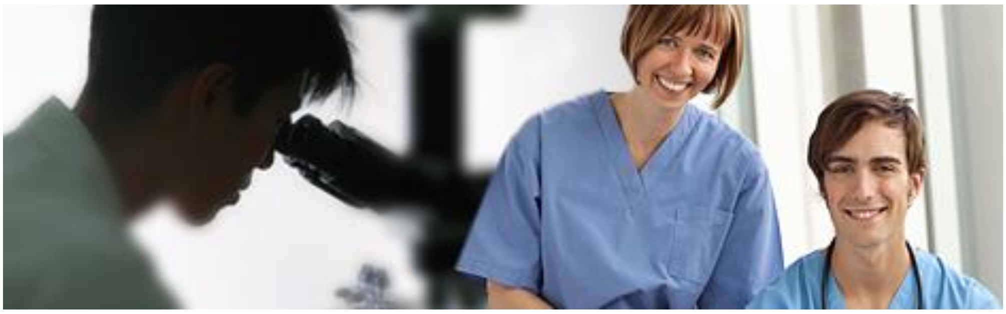

The Joanna Briggs Institute

Introduction

The Joanna Briggs Institute (JBI) is an international, membership based research and development organization within the Faculty of Health Sciences at the University of Adelaide. The Institute specializes in promoting and supporting evidence-based healthcare by providing access to resources for professionals in nursing, midwifery, medicine, and allied health. With over 80 collaborating centres and entities, servicing over 90 countries, the Institute is a recognized global leader in evidence-based healthcare.

JBI Systematic Reviews

The core of evidence synthesis is the systematic review of literature of a particular intervention, condition or issue. The systematic review is essentially an analysis of the available literature (that is, evidence) and a judgment of the effectiveness or otherwise of a practice, involving a series of complex steps. The JBI takes a particular view on what counts as evidence and the methods utilized to synthesize those different types of evidence. In line with this broader view of evidence, the Institute has developed theories, methodologies and rigorous processes for the critical appraisal and synthesis of these diverse forms of evidence in order to aid in clinical decision-making in health care. There now exists JBI guidance for conducting reviews of effectiveness research, qualitative research, prevalence/incidence, etiology/risk, economic evaluations, text/opinion, diagnostic test accuracy, mixed-methods, umbrella reviews and scoping reviews. Further information regarding JBI systematic reviews can be found in the JBI Reviewer's Manual on our website.

JBI Critical Appraisal Tools

All systematic reviews incorporate a process of critique or appraisal of the research evidence. The purpose of this appraisal is to assess the methodological quality of a study and to determine the extent to which a study has addressed the possibility of bias in its design, conduct and analysis. All papers selected for inclusion in the systematic review (that is – those that meet the inclusion criteria described in the protocol) need to be subjected to rigorous appraisal by two critical appraisers. The results of this appraisal can then be used to inform synthesis and interpretation of the results of the study. JBI Critical appraisal tools have been developed by the JBI and collaborators and approved by the JBI Scientific Committee following extensive peer review. Although designed for use in systematic reviews, JBI critical appraisal tools can also be used when creating Critically Appraised Topics (CAT), in journal clubs and as an educational tool.

© Joanna Briggs Institute 2017

THE JOANNA BRIGGS INSTITUTE

JBI Critical Appraisal Checklist for Qualitative Research

Reviewer______________________________________Date___________________________

Author ______________________________________Year_________Record Number______

		Yes	No	Unclear	Not applicable
1.	Is there congruity between the stated philosophical perspective and the research methodology?	☐	☐	☐	☐
2.	Is there congruity between the research methodology and the research question or objectives?	☐	☐	☐	☐
3.	Is there congruity between the research methodology and the methods used to collect data?	☐	☐	☐	☐
4.	Is there congruity between the research methodology and the representation and analysis of data?	☐	☐	☐	☐
5.	Is there congruity between the research methodology and the interpretation of results?	☐	☐	☐	☐
6.	Is there a statement locating the researcher culturally or theoretically?	☐	☐	☐	☐
7.	Is the influence of the researcher on the research, and vice- versa, addressed?	☐	☐	☐	☐
8.	Are participants, and their voices, adequately represented?	☐	☐	☐	☐
9.	Is the research ethical according to current criteria or, for recent studies, and is there evidence of ethical approval by an appropriate body?	☐	☐	☐	☐
10.	Do the conclusions drawn in the research report flow from the analysis, or interpretation, of the data?	☐	☐	☐	☐

Overall appraisal: Include ☐ Exclude ☐ Seek further info ☐

Comments (Including reason for exclusion)

____

____

____

© Joanna Briggs Institute 2017

Discussion of Critical Appraisal Criteria

How to cite: Lockwood C, Munn Z, Porritt K. Qualitative research synthesis: methodological guidance for systematic reviewers utilizing meta-aggregation. Int J Evid Based Healthc. 2015;13(3):179–187.

1. Congruity between the stated philosophical perspective and the research methodology

Does the report clearly state the philosophical or theoretical premises on which the study is based? Does the report clearly state the methodological approach adopted on which the study is based? Is there congruence between the two? For example:

A report may state that the study adopted a critical perspective and participatory action research methodology was followed. Here there is congruence between a critical view (focusing on knowledge arising out of critique, action and reflection) and action research (an approach that focuses on firstly working with groups to reflect on issues or practices, then considering how they could be different; then acting to create a change; and finally identifying new knowledge arising out of the action taken). However, a report may state that the study adopted an interpretive perspective and used survey methodology. Here there is incongruence between an interpretive view (focusing on knowledge arising out of studying what phenomena mean to individuals or groups) and surveys (an approach that focuses on asking standard questions to a defined study population); a report may state that the study was qualitative or used qualitative methodology (such statements do not demonstrate rigour in design) or make no statement on philosophical orientation or methodology.

2. Congruity between the research methodology and the research question or objectives

Is the study methodology appropriate for addressing the research question? For example: A report may state that the research question was to seek understandings of the meaning of pain in a group of people with rheumatoid arthritis and that a phenomenological approach was taken. Here, there is congruity between this question and the methodology. A report may state that the research question was to establish the effects of counselling on the severity of pain experience and that an ethnographic approach was pursued. A question that tries to establish cause-and effect cannot be addressed by using an ethnographic approach (as ethnography sets out to develop understandings of cultural practices) and thus, this would be incongruent.

3. **Congruity between the research methodology and the methods used to collect data**

   Are the data collection methods appropriate to the methodology? For example:

   A report may state that the study pursued a phenomenological approach and data was collected through phenomenological interviews. There is congruence between the methodology and data collection; a report may state that the study pursued a phenomenological approach and data was collected through a postal questionnaire. There is incongruence between the methodology and data collection here as phenomenology seeks to elicit rich descriptions of the experience of a phenomena that cannot be achieved through seeking written responses to standardized questions.

4. **Congruity between the research methodology and the representation and analysis of data**

   Are the data analyzed and represented in ways that are congruent with the stated methodological position? For example:

   A report may state that the study pursued a phenomenological approach to explore people's experience of grief by asking participants to describe their experiences of grief. If the text generated from asking these questions is searched to establish the meaning of grief to participants, and the meanings of all participants are included in the report findings, then this represents congruity; the same report may, however, focus only on those meanings that were common to all participants and discard single reported meanings. This would not be appropriate in phenomenological work.

5. **There is congruence between the research methodology and the interpretation of results**

   Are the results interpreted in ways that are appropriate to the methodology? For example:

   A report may state that the study pursued a phenomenological approach to explore people's experience of facial disfigurement and the results are used to inform practitioners about accommodating individual differences in care. There is congruence between the methodology and this approach to interpretation; a report may state that the study pursued a phenomenological approach to explore people's experience of facial disfigurement and the results are used to generate practice checklists for assessment. There is incongruence between the methodology and this approach to interpretation as phenomenology seeks to understand the meaning of a phenomenon for the study participants and cannot be interpreted to suggest that this can be generalized to total populations to a degree where standardized assessments will have relevance across a population.

© Joanna Briggs Institute 2017

6. **Locating the researcher culturally or theoretically**

   Are the beliefs and values, and their potential influence on the study declared? For example:

   The researcher plays a substantial role in the qualitative research process and it is important, in appraising evidence that is generated in this way, to know the researcher's cultural and theoretical orientation. A high quality report will include a statement that clarifies this.

7. **Influence of the researcher on the research, and vice-versa, is addressed**

   Is the potential for the researcher to influence the study and for the potential of the research process itself to influence the researcher and her/his interpretations acknowledged and addressed? For example:

   Is the relationship between the researcher and the study participants addressed? Does the researcher critically examine her/his own role and potential influence during data collection? Is it reported how the researcher responded to events that arose during the study?

8. **Representation of participants and their voices**

   Generally, reports should provide illustrations from the data to show the basis of their conclusions and to ensure that participants are represented in the report.

9. **Ethical approval by an appropriate body**

   A statement on the ethical approval process followed should be in the report.

10. **Relationship of conclusions to analysis, or interpretation of the data**

    This criterion concerns the relationship between the findings reported and the views or words of study participants. In appraising a paper, appraisers seek to satisfy themselves that the conclusions drawn by the research are based on the data collected; data being the text generated through observation, interviews or other processes.

© Joanna Briggs Institute 2017
